# The Overuse of the Emergency Department at a Multidisciplinary Pain Clinic by Patients with Chronic Pain: A Cross-Sectional Study

**DOI:** 10.7759/cureus.33757

**Published:** 2023-01-13

**Authors:** Filipa Felix, Elsa Oliveira, Sofia C Silva, Luís Agualusa

**Affiliations:** 1 Anaesthesiology, Hospital Pedro Hispano, Matosinhos, PRT; 2 Pain Medicine, Hospital Pedro Hispano, Matosinhos, PRT; 3 Pharmacology and Therapeutics, Hospital Pedro Hispano, Matosinhos, PRT

**Keywords:** pain clinic, emergency department visits, emergency department visit rates, emergency department, pain management, overusers, prescription opioid, multidisciplinary pain clinic, chronic pain

## Abstract

Introduction

Pain represents up to 78% of emergency department (ED) appointments, and an average of 16% of patients that consume ED resources have chronic pain. ED overuse could be an indicator of poor pain management. We are not aware of any study that has ever been carried out to understand the incidence of patients followed up at a multidisciplinary pain clinic (MPC) who overuse the ED. We aim to characterize patients in our MPC who overuse the emergency department, comprehend our percentages, and develop effective methods to reduce these numbers in the near future.

Materials and methods

We reviewed the medical records of patients observed in our MPC in 2019, selected patients with more than six ED visits from 2019 to 2021, and registered their ED visit diagnosis and evolution. We followed up on these patients and characterized them according to demographic aspects, chronic pain diagnostics, comorbidities, chronic medication, number of chronic pain department appointments, and patients that underwent invasive pain treatment.

Results

In 2019, 1892 patients were evaluated at our MPC, and only 1% were classified as overusers of the ED. The average number of episodes per patient was 10 in 2019, seven in 2020, and four in 2021. 70% of episodes were due to pain, and 94% were discharged immediately. The majority were women, and 69% were under the age of 69. Seventy-three percent had psychiatric disorders, and 95% and 89% were medicated with opioid and antidepressant medication, respectively, prior to the ED evaluation. Chronic primary pain was the most common diagnosis (47%), followed by chronic secondary musculoskeletal pain (21%). In 2019, most of these patients had only one appointment at our MPC, and in 2021, 79% had no appointments at all.

Conclusion

Our findings emphasize the particularities of patients with chronic pain who are followed in an MPC and misuse the ED. We observe the predominance of middle-aged people, which raises concerns about the impact of chronic pain in the active population. Issues like the predominance of patients with a diagnosis of primary chronic pain, psychiatric disorders, and being polymedicated with antidepressants and opioids are also a concern. We also realized that a high percentage of patients who overuse EDs lost follow-up at MPC over the past three years, which may reinforce the idea that they were misguided in the treatment of their chronic pain.

We recognized the need to improve teamwork with primary care in the follow-up of these patients as well as sensitize emergency service professionals to refer this patient rather than medicate acutely so that follow-up could be carried out in the appropriate places to reduce the percentage of ED overuse.

## Introduction

Chronic pain is a common, complex, and distressing problem that has a significant impact on society and individuals [1]. It is defined as pain that persists for more than three to six months, or beyond normal healing time, and affects between 19 and 30% of the population in Europe [2-3].

Up to 78% of all emergency department (ED) visits have been pain-related, and chronic pain accounts for 10%-16% of these visits [4]. Patients with chronic pain who visit the emergency department have a variety of medical and psychosocial issues, including long-term opioid therapy [4].

Since the ED is not considered an appropriate setting for chronic pain management and ED visits cost about five times as much as a general practice visit, their use for non-urgent medical conditions such as chronic pain puts the health care system's resources under great strain [2]. The most common reason for which chronic pain patients visit the ED is an inability to cope with pain, which has been linked to increased pain, depression, and an inability to adapt psychologically [5].

Maladaptive pain coping strategies, such as pain catastrophizing, not only lead to disability but also increased pain intensity, decreased quality of life, and increased use of acute care services [6].

Chronic pain management is beyond the scope of an intervention in the ED [2]. Healthcare providers in this setting have limited time and incomplete medical and medication histories to identify and prioritize the relative contributions of medical issues, mental health issues, and substance dependence to the complaint of chronic pain. Furthermore, prescribing opioids without a complete understanding of the patient's history may increase the risk of adverse drug-related events and complications, including death [7-8].

Although the definition of ED overuse is still not entirely clear, we consider it a strong indicator of poor pain management in patients with chronic pain [2]. In our study, we set a cutoff of six or more urgent episodes to define it.

Following the premise that overuse of emergency services is an indicator of poor management of chronic pain, especially in patients followed up in dedicated units, in this study we aim to identify the incidence of ED overusers, characterize these patients, and identify the factors that contribute to their ED presentation. We intend that these aspects will allow us to create, in the near future, a good foundation to evaluate the effectiveness of intervention in our multidisciplinary pain clinic (MPC) for the reduction of ED overuse.

## Materials and methods

To identify ED overusers and determine the incidence of ED overuse, we selected all patients with consultations at our MPC from 2019 and followed their attendance at the emergency department for three years consecutively.

Study design and time period 

This was a cross-sectional study consisting of a health record review of the list of patients with medical appointments at the MPC at Hospital Pedro Hispano, Matosinhos, Portugal, in 2019. This study was approved by the institutional research ethics board, and all informed consent was requested and approved. (IRB approval number: 141 I CES I JAS (Ref 61 ICLPSI I 2021)).

Setting and Population

The study was conducted at an MPC in the north of Portugal. We chose all patients who had six or more ED visits during the year from the 1892 patients who still had one appointment at our center in 2019. 

Procedures

We obtained the list of patients with six or more ED visits during 2019 from the hospital's administrative database. Each ED visit was classified as pain-related, trauma-related, or other. We recorded the total number of ED visits in 2019, 2020, and 2021, the Manchester sorting grade, the post-discharge destination, as well as the diagnosis for ED visits. The following data were collected: age, gender, address, chronic pain classification (according to the International Classification of Diseases, 11th Revision (ICD-11)), comorbidities, chronic medication, the total number of appointments at the chronic pain department, and the patients that underwent invasive pain treatment.

Statistical analysis 

We used IBM Statistical Package for the Social Sciences (SPSS) software to analyze the data. Descriptive and univariate analyses were performed. Continuous variables are presented as means and standard deviations (SDs), and categorical variables are presented as percentages and counts.

## Results

Among the 1892 patients who were evaluated in our MPC, only 19 patients (1% of the entire population) had six or more ED visits during 2019. This group had 409 emergency episodes during 2019, 2020, and 2021, and the average number of episodes per patient was 10 in 2019, seven in 2020, and four in 2021. The main diagnosis in the ED was pain (70%) and 94% were discharged immediately without any intervention. On the Manchester screening, 44% of patients were classified as yellow, 36% as orange, and 18% as green. All events resulted in 94% of patients being discharged, 5% being hospitalized, and 1% being referred to medical specialty areas.

Characterizing the overuser group, 80% were female, and the mean age was 65. According to their age, 69% of these patients were less than 69 years old. All patients had several chronic diseases and were on chronic medication. At 73%, psychiatric disorders were the most prevalent disease, and cardiac pathologies such as heart failure and ischemic disease, in parallel with respiratory pathologies such as obstructive sleep apnea syndrome, chronic obstructive pulmonary disease, and asthma, were the second and third most frequent diseases, respectively. We also discovered that 21% of patients who overused the emergency department had an oncological disease. The main etiological diagnoses for why these patients are followed up in an MPC, according to the ICD-11 classification of chronic pain, are primary chronic pain (47%) and musculoskeletal pain (21%). Also, chronic cancer-related pain, as well as chronic neuropathic pain and chronic postsurgical or posttraumatic pain, were other prevalent diagnoses, each with 11. 95% of the overusers were medicated with opioids prior to the emergency episode, and 33% were medicated with both weak and strong opioids. Relatively to the antidepressant medication, 89% of these patients were taking this kind of medication, and we also found that five patients were taking simultaneously two different types of antidepressants and that two patients were taking simultaneously five types.

The mean and standard deviation of ED overuse appointments in our center during 2019 were 1.16 ± 0.37, and only one patient underwent invasive procedures as part of a multimodal approach. In 2020, eight of these patients had no pain unit appointments, and the remaining patients were only followed up on two occasions. In 2021, the number of patients who had no follow-up in our MPC rose to 15, and only one had two appointments that year. In the end, there were two patients that were discharged from our MPC and three patients that passed away.

In the following table, we characterize the group of patients who overused the ED in 2019, 2020, and 2021.

**Table 1:** Characteristics of 19 patients who visited the emergency department (ED) too frequently for chronic pain * Patients had more than one comorbidity ICD-11: International Classification of Diseases 11th Revision

Characteristics	N	%
Gender		
Male	4	21
Female	15	79
Age (years)		
40-49	6	32
50-59	2	11
60-69	5	26
70-79	2	11
80-89	4	21
Comorbidities*		
Psychiatric	14	73^*^
Cardiac	9	52^*^
Respiratory	8	42^*^
Metabolic	7	37^*^
Oncologic	4	21^*^
Chronic pain classification (ICD-11)		
Chronic primary pain	9	47
Chronic cancer-related pain	2	11
Chronic secondary musculoskeletal pain	4	21
Chronic neuropathic pain	2	11
Chronic post-surgical or post-traumatic pain	2	11
Opioid medication	18	95
Weak opioid	11	58
Strong opioid	1	5
Both	6	32
Antidepressive medication	17	89
1 type	9	47
2 types	5	26
3 types	0	0
4 types	0	0
5 types	2	11
Chronic pain appointments		
2019		
0	0	0
1	16	84
2	3	16
2020		
0	8	42
1	6	32
2	5	26
2021		
0	15	79
1	3	16
2	1	5
ED visits		
2019	10	
2020	7	
2021	4	

## Discussion

Pain is one of the main reasons that leads patients to the emergency department, where poorly controlled or acute chronic pain accounts for 16% of the events. [3]

The percentages of patients with chronic pain who overuse the ED are known, but the incidence of ED overusers in units dedicated to the treatment of chronic pain is still unknown.

Since clinicians in emergency services do not have enough time to explore the management of chronic pain effectively and safely, the use of this service among this population brings an unnecessary use of resources and serious risks for the patients. [4]

We believe that misuse of the emergency department is an indicator of poor pain management, whether in terms of insufficiently treated pain or the inability of MPC to respond to these patients.

According to our data, only 1% of the 1892 patients evaluated in our MPC during 2019 were considered to have overused the ED. This percentage is most likely the result of a multimodal approach to the contribution of biological, psychological, and social or environmental factors to pain problems, which is critical in the management of chronic pain patients. Pain can have various etiologies, from physical to emotional, so a merely physical approach is clearly ineffective. We are also a center that favors targeted invasive procedures, implying a redoubled effort in carrying out the correct chronic pain diagnoses to obtain success with the invasive techniques. In our study population, on average, one in two patients was submitted to invasive procedures, but in the overuser group, the procedures were carried out on only one in every 20 patients, approximately. These results corroborate the importance of regional invasive techniques in chronic pain management, which may be one of the reasons for the low number of overusers in our center.

The combination of these measures and the open channel of communication with our MPC are probably responsible for the success of the treatment of these patients and the lower recurrence of ED visits.

Regarding demographic characteristics, these 19 patients were characterized, and they followed the descriptions found in the literature, with a predominance of female gender patients and the sixth decade of life. Regarding age groups, we found a predominance of ages below 69 years, with a higher incidence in patients between 40 and 49 and 60 and 69 years. Other aspects, such as work absence at this age and the socioeconomic impact of this illness behavior, will reflect this fact. The predominance of older ages, above 80 years, on the other hand, likely reflects the presence of severe musculoskeletal structural disease unresponsive to pharmacological therapies, as well as the difficulty of articulation in this age group when contacting MPC either by phone or e-mail.

The main diagnosis was primary chronic pain, given the absence of a well-defined etiological factor and the increasing challenge of managing these situations. In fact, an approach other than pharmacological treatment is truly required for this type of diagnosis. Associated with this major chronic pain diagnosis, a large percentage of patients have a history of psychiatric pathology, and almost 90% are chronically medicated with antidepressants. This factor corroborates what the literature shows: that these patients are unable to create strategies to cope with pain, leading to the recrudescence of pain, depression, and an inability to adapt psychologically, and consequently the recurrence of medical care, especially in the ED. [5] Moreover, there are a considerable number of patients (41%) who take more than one type of antidepressant. This probably reflects the complexity of psychiatric disorders or the difficulty of managing these kinds of patients.

Almost 100% of patients were medicated with opioids and a higher percentage with both weak and strong opioids. This brings us to the question about the use of opioids for the treatment of non-cancer pain, especially in situations of chronic primary pain, whose treatment is more dependent on a holistic approach. According to the literature, patients with primary pain syndromes may be less responsive to opioids due to higher endogenous opioid levels and more susceptible to the worsening of hyperalgesia by opioids, so this kind of treatment is clearly advised against. Furthermore, the potential for dangerous side effects, physical tolerance, misuse, or addiction increases the risk of their use in the treatment of this kind of pathology. [9] 

In the sequence of our analysis, during the three years under study, patients who overused the ED lost follow-up at the MPC, and in 2021, only four (20%) patients were consulted. Because these patients were not well followed up on in the MPC, they had to visit the ED more frequently, and the lack of follow-up meant that they continued to overuse the ED in the years that followed. In these cases, ED physicians should be educated to refer these patients back to primary health care or to MPC, where the achievement of pain control can be most effective and lasting, and to avoid prescribing opioids indiscriminately.

Primary health care has a key role in managing chronic pain patients, mainly by maintaining follow-up, prescribing chronic medication, or facilitating referrals to specialist departments. However, literature shows that chronic pain patients do not feel confident about managing their pain with a primary health assistant, and general practitioners do not feel secure in the approach to more complex chronic pain situations. [2,10]

In the Manchester screening, we observed that although most episodes were classified as yellow or orange, a great proportion of patients were discharged after receiving intravenous medication or an opioid prescription. These episodes can also be avoided by articulating with the MPC. The ease of accessing the emergency room and the administration of drugs with a quick effect on reducing pain are identified as two of the main reasons that lead chronic pain patients to the emergency room. [1] 

Limitations

We did not statistically study the impact of the SARS-CoV-2 pandemic on reducing emergency department utilization, which significantly reduced the influx of patients and probably influenced the results for 2020 and 2021. Another limitation of the study is the restriction of the evaluation of emergency episodes to our institution, knowing the possibility of patients having resorted to other institutions.

The lack of a control group of chronic pain patients without follow-up in our MPC makes comparing our population to the general population difficult. However, it allows us to build a fundamental knowledge base that could enable us, in the future, to design an effective study of our approach. Despite being in its early stages, our work has significance and can serve as a jumping-off point for others because there is no comparable study in the literature.

## Conclusions

Noting the dynamics of the ED overuse group at our center, it turns out that chronic pain is a public health problem with an important economic impact. It is primordial to improve collaboration with primary health care in the follow-up of these patients, as well as sensitize emergency service professionals to refer these patients instead of medicating them acutely. This will allow for follow-ups to be carried out in the appropriate places and the reduction of ED overuse. This strategy will minimize the number of patients who get lost in the system, as happened with the pandemic, and will avoid indiscriminate prescriptions of opioids in the ED.

The importance of realizing that patients overuse resources is crucial to reducing costs and improving patient safety. We believe that the development of more studies on this topic is essential to improving the quality of care provided to patients with chronic pain.

## References

[1] Todd KH, Cowan P, Kelly N, Homel P (2010). Chronic or recurrent pain in the emergency department: national telephone survey of patient experience. West J Emerg Med.

[2] Mills SE, Nicolson KP, Smith BH (2019). Chronic pain: a review of its epidemiology and associated factors in population-based studies. Br J Anaesth.

[3] Poulin PA, Nelli J, Tremblay S (2016). Chronic pain in the emergency department: a pilot mixed-methods cross-sectional study examining patient characteristics and reasons for presentations. Pain Res Manag.

[4] Shergill Y, Rice D, Smyth C (2020). Characteristics of frequent users of the emergency department with chronic pain. CJEM.

[5] Peres MF, Lucchetti G (2010). Coping strategies in chronic pain. Curr Pain Headache Rep.

[6] Meyer K, Tschopp A, Sprott H, Mannion AF (2009). Association between catastrophizing and self-rated pain and disability in patients with chronic low back pain. J Rehabil Med.

[7] Dhalla IA, Mamdani MM, Sivilotti ML, Kopp A, Qureshi O, Juurlink DN (2009). Prescribing of opioid analgesics and related mortality before and after the introduction of long-acting oxycodone. CMAJ.

[8] Dixon WJ, Fry KA (2011). Pain recidivists in the emergency department. J Emerg Nurs.

[9] Krčevski Škvarč N, Morlion B, Vowles KE (2021). European clinical practice recommendations on opioids for chronic noncancer pain - Part 2: Special situations. Eur J Pain.

[10] Johnson M, Collett B, Castro-Lopes JM (2013). The challenges of pain management in primary care: a pan-European survey. J Pain Res.

